# Improved mechanical strength, proton conductivity and power density in an ‘all-protonic’ ceramic fuel cell at intermediate temperature

**DOI:** 10.1038/s41598-021-98987-6

**Published:** 2021-09-29

**Authors:** Abul K. Azad, Abdalla M. Abdalla, Ahmed Afif, Atia Azad, Shammya Afroze, Azam Che Idris, Jun-Young Park, Mohammad Saqib, Nikdalila Radenahmad, Shahzad Hossain, Iftakhar Bin Elius, Md. Al-Mamun, Juliana Zaini, Amer Al-Hinai, Md. Sumon Reza, John T. S. Irvine

**Affiliations:** 1 Faculty of Integrated Technologies, Universiti Brunei Darussalam, JalanTungku Link, Gadong, BE1410 Brunei Darussalam; 2 Mechanical Engineering Department, Faculty of Engineering, Suez Canal University, Ismailia, 41522 Egypt; 3 School of Chemistry, University of St Andrews, Fife, KY16 9ST UK; 4 Department of Nanotechnology and Advanced Materials Engineering, HMC & Green Energy Research Institute, Sejong University, Seoul, 143-747 Republic of Korea; 5 Institute of Nuclear Science and Technology, Bangladesh Atomic Energy Commission, Savar, Dhaka, Bangladesh; 6 Sustainable Energy Research Center, Sultan Qaboos University, Muscat, Oman

**Keywords:** Energy science and technology, Materials science, Physics

## Abstract

Protonic ceramic fuel cells (PCFCs) have become the most efficient, clean and cost-effective electrochemical energy conversion devices in recent years. While significant progress has been made in developing proton conducting electrolyte materials, mechanical strength and durability still need to be improved for efficient applications. We report that adding 5 mol% Zn to the Y-doped barium cerate-zirconate perovskite electrolyte material can significantly improve the sintering properties, mechanical strength, durability and performance. Using same proton conducting material in anodes, electrolytes and cathodes to make a strong structural backbone shows clear advantages in mechanical strength over other arrangements with different materials. Rietveld analysis of the X-ray and neutron diffraction data of BaCe_0.7_Zr_0.1_Y_0.15_Zn_0.05_O_3−δ_ (BCZYZn05) revealed a pure orthorhombic structure belonging to the *Pbnm* space group. Structural and electrochemical analyses indicate highly dense and high proton conductivity at intermediate temperature (400–700 °C). The anode-supported single cell, NiO-BCZYZn05|BCZYZn05|BSCF-BCZYZn05, demonstrates a peak power density of 872 mW cm^−2^ at 700 °C which is one of the highest power density in an all-protonic solid oxide fuel cell. This observation represents an important step towards commercially viable SOFC technology.

## Introduction

As the standard technologies based on fossil fuels cannot satisfy the growing demand for energy, the future lies in the implementation of efficient and environmentally friendly technologies to produce electricity, such as hydrogen energy and fuel cells^1–3^. During the past few decades, fuel cells, especially solid oxide fuel cells (SOFCs), have attracted significant attention due to their high efficiency, low pollution and low environmental impact at high working temperatures (700–1000 °C)^4^. Yttria Stabilized Zirconia (YSZ) is a state-of-the-art electrolyte because it possesses adequate oxide-ion conductivity (∼ 0.13 Scm^−1^ at 1000 °C) and shows desirable phase stability in both oxidizing and reducing atmospheres^5^. However, its high operating temperature leads to high system costs, cell degradation, low material compatibility and dissatisfactory durability^6,7^. To reduce and overcome these problems, protonic ceramic fuel cells (PCFCs) have been introduced. PCFCs operate in an intermediate temperature (IT) range (400–700 °C) due to the higher mobility of proton ions than oxygen ions^8–12^. Duan et al. recently proved that PCFCs can be efficiently used as reversible protonic ceramic electrochemical cells (RePCECs)^13^. Fop et al. observed both oxide ion and proton conductivity in disordered hexagonal perovskites^14^. Over the last few decades, numerous materials, especially electrolyte materials, have been explored to obtain better performance with lower activation energies in IT range fuel cell applications^15–22^. Recently, Haile et al. proved that pulsed laser-deposited PrBa_0.5_Sr_0.5_Co_1.5_Fe_0.5_O_5+δ_ cathodes show very good performance and long-term stability with a PCFC electrolyte^23^.

Due to the highly resistive nature of its grain boundaries, BaZrO_3_ has a lower conductivity but higher chemical and thermal stability under both H_2_O and CO_2_ than BaCeO_3_. However, BaCeO_3_–BaZrO_3_ doped with yttrium along with different Ce and Zr contents exhibits high conductivity with good chemical stability and high cell performance^24–27^. Using ZnO as a sintering additive allows a reduction in the high sintering temperature and remarkable improvement in the density, conductivity and stability^28–30^. Highly dense BZY electrolyte can also be obtained by solid state reactive sintering^31^.

A recent review from Irvine et al. discussed the processes occurring at the interface and ways to control the structure at the nanoscale for high performance and durability in solid oxide cells (SOCs)^32^. The high operating temperature of SOCs gives significant constraints for material selection of electrodes, electrolytes and interconnects. All materials should be nonreactive and have a matching thermal expansion coefficient. Electrodes must be redox stable and porous and have mixed ionic and electronic conductors; electrolytes and interconnects must be gas impermeable and redox stable. An ideal microstructure is crucial to optimise contacts between the electrolyte and the electrodes to be mechanically, chemically, dimensionally and thermally stable during operation. Durability depends on the ability to withstand pressure or damage, which is also highly dependent on the hardness or elastic modulus of the fuel cell. Duan et al.^33^ showed the long-term durability and fuel flexibility of protonic ceramic fuel cells. This highlights the potential of the technology for commercial applications but without observing the mechanical properties of the fuel cells themselves. Another very interesting and important property of PCFCs is that they do not suffer fuel dilution in the way oxide ions conducting SOFCs do since water forms on the cathode side. Since the fuel dilution on the anode side does not decrease the Nernst potential, the PCFC can maintain a higher operation voltage, particularly for high fuel utilization.

The mechanical properties are one of the most important and critical characteristics to be considered due to the different thermal expansion coefficients of the constituting materials and specific work environments. Nanoindentation/indentation methods are an attractive approach to determine the hardness and Young’s modulus, which has a direct relation with durability. To determine the hardness and elastic modulus, Oliver Pharr analysis^34^ is commonly used when *h*_*f*_*/h*_*m*_ (final indentation depth/peak load indentation depth) is less than 0.7. When the pile-up effect is observed, the relationship between the hardness and work of indentation can be written as $H=\frac{k{P}_{m}^{3}}{9{W}^{2}}$, where P is the maximum load, k is a constant that depends on the geometry of the indenter, and W is the total work or plastic work.

**Figure 1:**
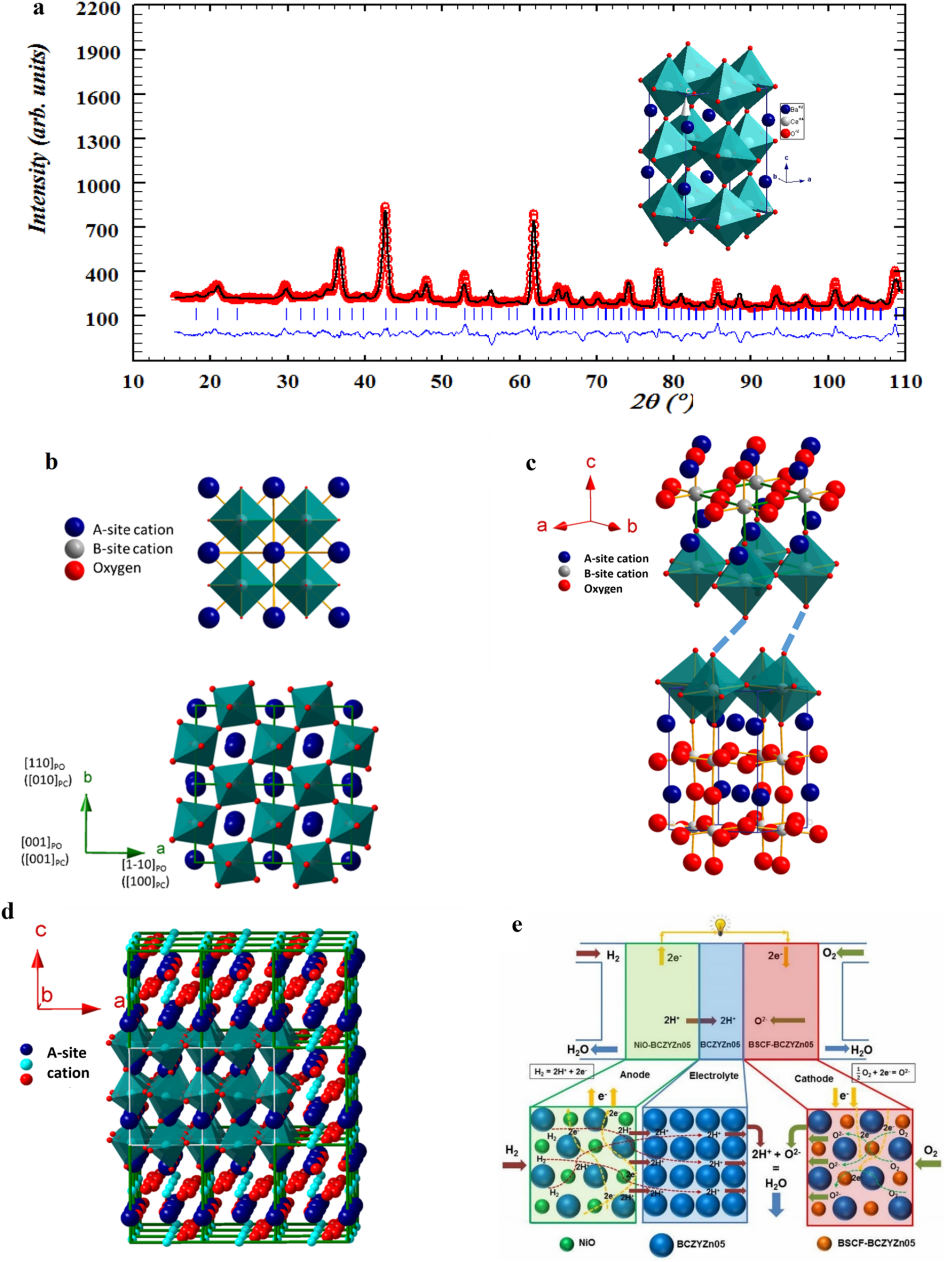
Crystal structure determination and atomic arrangements. (**a**) Rietveld refinement of the neutron diffraction data of BCZYZn05 polycrystalline ceramic powder in orthorhombic symmetry in the *Pbnm* space group. The unit cell parameters are related to the ideal primitive cubic perovskite cell as *a* ≈ √*2a*_*p*_*, b* ≈ √*2a*_*p*_ and *c* ≈ *2a*_*p*_ (*a*_*p*_ ≈ 3.96 Å is the unit cell parameter of ideal primitive perovskite). Schematic 3D structure is shown in insert. (**b**) c-axis view of cubic (space group *Pm-3m*) BSCF cathode and orthorhombic BCZYZn05 electrolyte. In Glazer’s notation, the rotation of oxygen octahedra in this cubic and orthorhombic space group can be described as *a*^0^*a*^0^*a*^0^ and *a*^*−*^*a*^*−*^*c*^+^, respectively. In the orthorhombic case, the rotation is in-phase around the [001]*po* axis and out-of-phase around the [1–10]_*po*_ axis, which indicates that the octahedral distortion in the BCZYZn05 structure will be visual along the [001]_*po*_ direction. (**c**) The dotted line represents the mismatch between the cubic and pseudocubic (orthorhombic) structures. (**d**) When the same material was used in the cathode|electrolyte|anode, no shifts of the oxygen atoms in the interface occurred, and the arrangement created a strong structural backbone for solid oxide fuel cells. (**e**) Schematic diagram of the all-protonic cell, which shows the proton conducting path and formation of water at the cathode side. Atomic structure of anode, electrolyte and cathode is shown in the lower part. NiO and Ba_0.5_Sr_0.5_Co_0.8_Fe_0.2_O_3_ powders were mixed with BCZYZn05 electrolyte powder to make anode and cathode, respectively.

Atomic structural matching is also an important property to consider for ceramic electrode/electrolyte interface durability and performance. The interface of atoms is dynamic and evolving in SOC subsystems, which has been proven theoretically and experimentally. Recent reports demonstrate that this dynamic nature may enhance durability and performance^32^. To understand and control the independence between interface structure, functionality, electrochemistry, performance and durability, different approaches were tried. Octahedral distortions have very close links to functional properties and durability in perovskite oxides, ABO_3_, which consists of three-dimensional corner-sharing BO_6_ octahedra in the perovskite lattice (Fig. 1b). Atomic structural matching improves the mechanical as well as electrochemical properties for intermediate- and high-temperature applications but has been omitted in most of the experimental, theoretical and review work carried out previously. Aso et al.^35^ discussed the effect of octahedral distortions in the heterointerface of perovskite oxides on the atomic level. The oxygen arrangements around the heterointerface, due to the octahedral mismatch, play a critical role in epitaxial strain accommodation in perovskite heterostructures. The matching thermal expansion coefficient among the anode/electrolyte/cathode is also extremely important for fuel cell durability. It can maximize the use of the same material due to their structural, thermal and behavioural similarity.

We explored the orthorhombic perovskite BaCe_0.7_Zr_0.1_Y_0.15_Zn_0.05_O_3−δ_ (BCZYZn05) electrolyte, which exhibits a high power density with high conductivity and better mechanical properties when BCZYZn05 was used in all parts of the ceramic cell (anode|electrolyte|cathode). Ba_0.5_Sr_0.5_Co_0.8_Fe_0.2_O_3_ (BSCF) cathode material^6^ was mixed with BCZYZn05 in a 3:2 weight percent to obtain high performance and durability. Atomic scale matching is one of the most important criteria for the improvement of fuel cell durability and mechanical strength which was rarely considered before where NiO or BSCF was used as oxide ion conductor electrodes. The main idea is to make a cell where same material can be used in anode, electrolyte and cathode, which will make the structural backbone of the cell. A single-cell NiO-BCZYZn05|BCZYZn05|BSCF-BCZYZn05 displays a power density of 872 mW cm^−2^ at 700 °C with the addition of an anode functional layer (AFL) using the drop-coating electrolyte deposition method. The performance may be further improved by controlling the preparation process and microstructure of the electrodes. Our results prove that using a single structured material in the anode, electrolyte and cathode to create a strong backbone of the cell is beneficial to improve the mechanical strength as well as performance and durability.

## Results and discussion

A single-phase material with nominal composition BaCe_0.7_Zr_0.1_Y_0.15_Zn_0.05_O_3−δ_ was obtained by solid-state reaction at 1400 °C for 10 h (Supplementary Information 2.1). The density of the BCZYZn05 electrolyte was found to be about 98% of the theoretical density. Rietveld refinement of the neutron diffraction data of the perovskite-type ceramic powder BCZYZn05 shows a well-formed orthorhombic structure in the *Pbnm* space group. The unit cell parameters were found to be *a* = 6.099(2) Å, *b* = 6.091(2) Å and *c* = 8.611(2) Å. Neutron powder diffraction (NPD) is a powerful technique that is used for its ability to detect light molecular weight elements such as hydrogen and oxygen, which shows additional peaks and, consequently, changes to the crystalline system. Determination of accurate oxygen position is crucial to find the octahedral tilting in the perovskite structure. BO_6_ octahedral tilting is common and is important not only for structural stability but also for its relation with physical properties ranging from ionic conductivity, electronic and magnetic properties, and metal–insulator transitions to improper ferroelectricity. Static and/or dynamic Jahn–Teller distortion of BO_6_ octahedra arises when two axial atomic bonds are shorter or longer than those of the equatorial bonds. The distortion affects the electrical, physical and mechanical properties. The difference in the neutron powder diffraction (NPD) pattern and X-ray powder diffraction (XRPD) pattern can be realized from Fig. 1a. NPD shows more Bragg reflections with good intensity due to the sensitivity for oxygen positions. As shown in Fig. 1b, BCZYZn05 crystallizes in an orthorhombic crystal structure with a *Pbnm* space group (*√2a*_*p*_ × *√2a*_*p*_ × *2a*_*p*_) that shows in-phase octahedral rotation around the [001]_*po*_ axis and out-of-phase rotation around the [1–10]_*po*_ axis, which can be described as *a*^*−*^*a*^*−*^*c*^+^ in the Glazer notation^36^. Therefore, the 3D images projected along the [001]_*po*_ direction are suitable to investigate octahedral distortions in the BSCF/BCZYZn05 (cubic/orthorhombic) heterostructure. In cubic *Pm-3m*, the rotations in all three directions are the same and can be described as *a*^0^*a*^0^*c*^0^ in Glazer notation (as shown in Fig. 1c). The rotations in *po* (perovskite orthorhombic) and *pc* (perovskite cubic) are related as [001]_*pc*_ → [001]_*po*_, [010]_*pc*_ → [110]_*po*_ and [100]_*pc*_ → [1–10]_*po*_. This is because of the large difference in the lattice parameters (BSCF; *a*_*pc*_ = 3.99 Å, BCZYZn05; *a*_*po*_ = 6.12 Å) as well as oxygen octahedral tilt angle *θ*, where *θ*_*BSCF*_ = 180°, *θ*_*BCZYZn05*_ = 175.130(9)° for B-O1-B and 160.24(9)° for B-O2-B. The atomic interface heterostructure explains how the structural distortions arise from structural mismatch, which is not only the effect of lattice parameters but also octahedral tilting.

A small percentage of electrolyte material mixed with the cathode/anode can increase the strength of the structural backbone of the cell and thus increase the durability (Fig. 1d). A schematic diagram of a proton conducting SOFC is given in Fig. 1e to show the proton and electron migration path.

**Figure 2:**
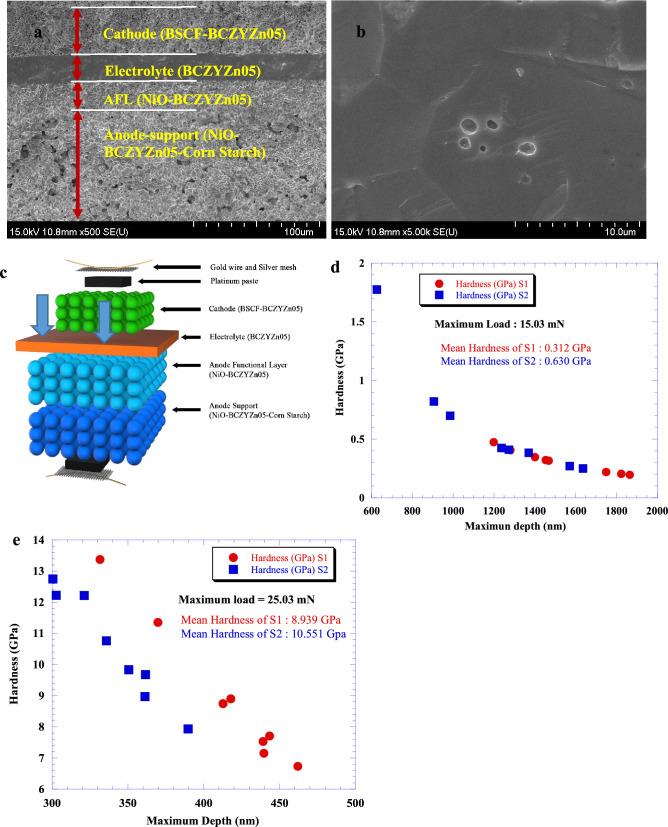
SEM analysis and hardness test. (**a**) Cross-sectional image of the single cell indicating the porosity difference in the anode and cathode and electrolyte. The electrolyte is the densest part of the cell and is approximately 26 μm thick. (**b**) Cross-section of the electrolyte to observe the grain size and boundaries. (**c**) Schematic diagram of nanoindentation on the cathode and electrolyte surface. Due to the higher density, the electrolyte is the hardest part of the cell. (**d**) Comparison of the hardness of the cathode between two cells without mixing BCZYZn05 with BSCF (S1) and with mixing BCZYZn05 (40%) with. BSCF (60%) (S2). (**e**) Comparison of hardness between two electrolytes: Ba_0.9_Sr_0.1_Ce_0.5_Zr_0.35_Y_0.1_Sm_0.05_O_3−δ_^40^ (S1) and BCZYZn05 (S2).

High density and high ionic conductivity are two of the most important criteria for an electrolyte for solid oxide fuel cells. A small percentage of Zn doping at the B-site^37^ or adding a sintering additive^38^ increases the density and performance of the electrolyte. Zn doping has advantages over Ni for better sintering, crystal structure and density. Ni doping suffers from unexpected electronic conduction in electrolytes with low OCVs, which might be due to electronic leakage related to the low transport number, fuel efficiency and power density^39^. The cross-sectional SEM image of NiO-BCZYZn05|BCZYZn05|BSCF-BCZYZn05 (see Fig. 2a) shows the density profile of the different parts of the cell. NiO-BCZYZn05 (60:40 wt%) anode-supported cells with the BCZYZn05 electrolyte and BSCF-BCZYZn05 cathode clearly show that the electrolyte was highly dense to stop gas crossover. The AFL was deposited between the NiO–BCZYZn05 anode support and BCZYZn05 electrolyte to reduce the difference in the shrinkage rate of both layers. The as-prepared AS shows a uniform porous structure with micro- and macro-pores. No delamination behaviour was found between the anode and electrolyte, indicating the good wettability of the anode and electrolyte. The BSCF-BCZYZn05 composite cathode also shows uniform micropores. The interaction phenomenon between the electrolyte and cathode was excellent after sintering at 1000 °C for 2 h which was observed from the cross-sectional SEM image of the cell (Fig. 2a). The electrolyte thickness was about 26.6(1) μm. To determine the density, phase and any impurities of the electrolyte materials, SEM was performed on the BCZYZn05 pellet sintered at 1400 °C for 10 h in air (Supplementary Document Fig. S2a,b). A cross-sectional image of the high-density electrolyte pellet is shown in Fig. 2b. No appreciable traces of impurities were discovered on the pellet surface or the cross-sectional area, which agrees with the XRD and NPD results. The relative density of BCZYZn05 was 98% (Supplementary Document, Table S1) and was calculated via the Archimedes method and neutron diffraction results. The average grain size of the sample was 4.7 μm.

The hardness of the cell can be calculated using the Oliver Pharr method^34^, which has the formula $H = ~\frac{{P_{m} }}{{24.56~h_{c}^{2} }}$, where *P*_*m*_ is the maximum load and *h*_*c*_ is the contact depth for a triangular pyramidal intender with an identical depth-to-area relationship. Taking into account the fact that elastic displacement occurs in both specimen *E* and Poisson’s ratio ν and the intender with elastic constants *E*_*i*_ and *ν*_*i*_, the reduced modulus *E*_*r*_ can be calculated by the following equation:

1 $$ \frac{1}{{E_{r} }} = ~\frac{{\left( {1 - \nu ^{2} } \right)}}{E} - ~\frac{{\left( {1 - \nu _{i}^{2} } \right)}}{{E_{i} }}. $$

Figure 2c shows the schematic atomic diagram of the cell and indentation points. The nanoindentation using a 15.03 mN load on 8 different points in the cathode surface shows that the mean hardness of the electrolyte mixed cathode (BSCF + BCZYZn05) was 102% higher than that without the electrolyte (BCZYZn05) mixed cathode (BSCF). This increase in mechanical strength is related to the atomic structural matching among anode, electrolyte and cathode for using same material to prepare them. The porosity of the cathode might be the same but mechanical strength increased a lot. The mean hardness of the BSCF + BCZYZn05 and BSCF cathodes was 0.63 GPa and 0.312 GPa, respectively (Supplementary Document Tables S3, S5). The maximum indentation depths were found to be 1635.2 nm and 1865.4 nm in the BSCF + BCZYZn05 and BSCF cathodes, respectively. Since the thickness of the cathode layers was more than 50,000 nm, the measured hardness effect was solely from the cathode and not from the cathode/electrode interface. The maximum indentation depth versus hardness of the two cathodes is compared in Fig. 2d. For the electrolytes, the hardness of BCZYZn05 was compared with a similarly dense electrolyte, Ba_0.9_Sr_0.1_Ce_0.5_Zr_0.35_Y_0.1_Sm_0.05_O_3−δ_ (BSCZYSm)^40^. The mean hardness of BCZYZn05 was approximately 18% higher than that of BSCZYSm (Fig. 2e). The maximum indentation depths using a 25.03 mN load on the electrolyte were 389.6 nm and 443.3 nm for BCZYZn05 and BSCZYSm, respectively (the thickness of the electrolytes was more than 25,000 nm). These results also prove that the electrolytes are much harder than the cathodes. Due to the different distances between test points and the interface, the energetic quantities have no linear relationships among the 8 test points.

**Figure 3:**
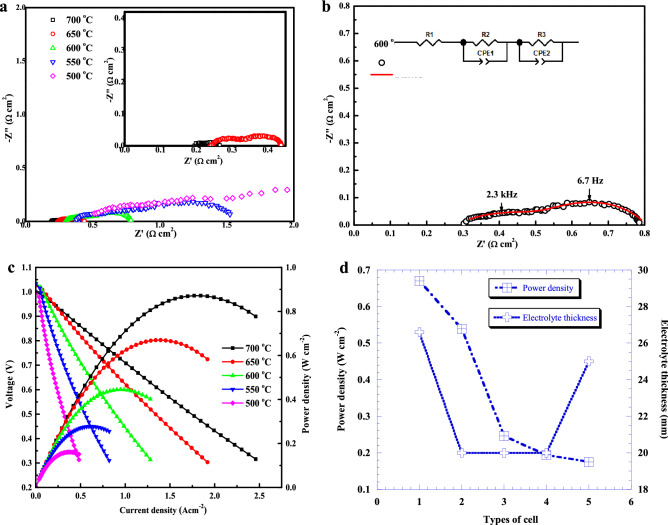
Electrochemical properties of NiO-BCZYZn05|BCZYZn05|BSCF-BCZYZn05 single cells. (**a**) Electrochemical impedance spectra collected at 500–700 °C in 50 °C intervals. (**b**) Fitted Nyquist impedance plot at 600 °C, which shows two semicircles; the equivalent circuit is shown in the insert. (**c**) *I–V* and power-density curves of the BCZYZn05-based fuel cell using humidified H_2_ (3% H_2_O) as the fuel and ambient air as the oxidant for the cathode at 500–700℃. (**d**) Comparison of power density and thickness of 4 similar proton conducting cells with the cell under study. The cells, electrolyte fabrication process and measured temperatures were as follows: 1. NiO-BCZYZn05|BCZYZn05|BSCF-BCZYZn05, drop coating, 650 °C (present study), 2. NiO-BCZY712|BCZY712|LSF-BCZY712, co-pressing, 650 °C^41^, 3. NiO-BZCYZn04|BZCYZn04|SSC-BZCYZn04, co-pressing, 600 °C^42^, 4. NiO-BZCYZn04|BZCYZn04|PBC-BZCYZn04, co-pressing, 600 °C^43^, 5. NiO-BZCYZn04|BZCYZn04|LSCF-BZCYZn04, tape casting, 600 °C^44^.

High electrocatalytic activity and ionic and electronic conduction are three essential functionalities for the electrochemically active region of fuel cell electrodes. These functionalities are extremely important aside from durability, cell stability, cell integration and mass transport. To illustrate the good performance of the single cell, electrochemical impedance spectra of the cell were obtained at various working temperatures. Typical Nyquist plots at different temperatures are shown in Fig. 3a. The high-frequency intercept on the real axis represents the ohmic resistance (R_ohm)_ of the cell, which mainly originates from the resistance of the electrolyte. The difference between the low-frequency and high-frequency intercepts with the real axis designates the interfacial polarization resistance (R_p_), which is mainly dominated by the electrode materials and microstructures^45^. The area-specific resistance (ASR) of a single cell was calculated (fitted) from the impedance spectra using EC lab software with an equivalent circuit model, where R_1_ is R_ohm_ and the sum of R_2_ and R_3_ is R_p_. Figure 3b presents the fitting profile of the obtained spectra with an equivalent circuit at 600 °C. The obtained frequencies for the first semicircle and second semicircle were 2.3 kHz and 6.7 Hz, respectively. The total conductivity (ionic plus electronic) values of the single cell were 2.3 × 10^–2^, 5.17 × 10^–2^, 0.13, 0.34 and 0.97 Scm^−1^ at 500, 550, 600, 650 and 700 °C, respectively. The bulk and total conductivities of the electrolyte material reached 9.23 × 10^–3^ and 7.61 × 10^–3^ under wet Ar conditions, where the activation energies were below 0.6 eV. Proton migration in this system was related to the incorporation of proton via dissociation of water into oxygen vacancies generated by acceptor doping of the host ceramic material^46^. Protons are introduced as point defects that become mobile as the temperature increases via the transport phenomenon known as the Grotthus mechanism^47^. This low activation energy makes fuel cells more proton conductive, affordable and practical. The R_ohm_ values were 0.196, 0.238, 0.269, 0.266 and 0.306 Ωcm^2^, and the R_p_ values were 0.07, 0.207, 0.543, 1.405 and 3.427 Ωcm^2^ at 700, 650, 600, 550 and 500 °C, respectively. Compared with previous results, both R_ohm_ and R_p_ values are lower than those of many BCZY-based cells^48^. The processing of materials, cell preparation and testing have strong effect on the cell performance, hence, it is difficult to compare the properties of different cells (for more information, please see Table S6 in the Supplementary File). Recently, An et al.^49^ obtained power density of 1.3 Wcm^−2^ in a 5 × 5 cm^2^ protonic ceramic fuel cell at 600 °C with R_ohm_ of 0.09 Ωcm^2^. Particularly, among the ‘all-protonic’ type cells ^41–44,50–52^, our present study has shown very promising results. The excellent performance of the present cell is due to the low cell resistances. The low R_ohm_ values should be attributed to the highly dense and thin electrolyte film. Current–voltage–power (IVP) with area-specific resistance curves of the as-fabricated NiO-BCZYZn05|BCZYZn05|BSCF-BCZYZn05 single cells with added AFL using 3% humidified H_2_ as the fuel and ambient air as the oxidant in the temperature range of 500–700 °C are presented in Fig. 3c. The observed peak power densities were 161, 278, 445, 670 and 872 mWcm^−2^ at 500, 550, 600, 650 and 700 °C, respectively. The cell performance was highly promising in comparison to similar cell performances, as shown in Fig. 3d (a detailed list of closely related proton conducting SOFCs is presented in the Supplementary File, Table S6). The total performance of the fuel cell depends not only on the materials but also on many other factors, such as processing, thickness, gas flow, and current collector. Therefore, it is difficult to understand from the comparison of different cell arrangements. The coking resistance and sulfur tolerance and thermal cycling stability are crucially important for fuel cell commercialization, particularly for applications requiring start/stop capability or for transient or variable loads. Duan et al. demonstrated and achieved excellent performance, robust and exceptional durability with direct operation on 11 different fuel streams without any modifications in the cell composition or architecture, many of which have not previously been studied in a PCFC^33^. Fuel cell performance can vary greatly depending on the processing of the materials for electrodes and electrolytes and their thickness. Figure 3d also compares the electrolyte thickness with the performance of 5 similar cells. There is no linear relationship between electrolyte thickness and performance. Even with a high thickness (26.6 μm), the BCZYZn05 electrolyte shows better performance than the others. The open circuit voltage (OCV) values of the cell were 0.998, 1.034, 1.037, 1.027 and 1.0 V at 500, 550, 600, 650 and 700 °C, respectively. This confirms the high density of the electrolyte.

In summary, the strategy of adding Zn to Y-doped barium cerate-zirconate was successful in decreasing the sintering temperature and creating a high-density electrolyte with high proton conductivity. The BCZYZn05 electrolyte material was investigated for IT-SOFCs in terms of conductivity, density and performance. XRD and neutron patterns of the electrolyte were measured and showed a pure phase with an orthorhombic structure. Moreover, an anode-supported single cell with the configuration NiO-BCZYZn05|BCZYZn05|BSCF-BCZYZn05 was fabricated and obtained a maximum power density of 872 mWcm^−2^ at 700 °C. Although the results demonstrate that BCZYZn05 is a promising proton-conducting electrolyte for intermediate-temperature solid oxide fuel cells due to its high output performance, further improvement can be achieved. This can be done by lowering the R_p_ values using better cathode materials and reducing the electrolyte thickness. Moreover, the high performance combined with good conductivity demonstrates that the BaCe_0.7_Zr_0.1_Y_0.15_Zn_0.05_O_3-δ-_based cell is a promising IT-SOFC with good mechanical strength, durability and performance.

## Methods

### Fabrication of the electrolyte

BaCe_0.7_Zr_0.1_Y_0.15_Zn_0.05_O_3−δ_ powders were synthesized via a solid-state reaction process from BaCO_3_ (99% purity, Merck, Germany), CeO_2_ (99% purity, Aldrich, China), ZrO_2_ (99% purity, Sigma-Aldrich, UK), Y_2_O_3_ (99.9% purity, Aldrich, China) and ZnO (99% purity, Merck, Germany). Stoichiometric amounts of selected materials were ball-milled in ethanol using zirconia balls for 24 h. The ball-milled materials were dried in an oven and calcined at 650 °C for 10 h, cooled down to room temperature (RT) and subsequently ground and palletized using a 15 mm diameter die under 20 MPa pressure. The palletized sample was sintered at 1000 °C for 10 h and cooled to RT. The pellet was again ground, palletized and sintered at 1200 °C for 10 h and finally at 1400 °C for 10 h. X-ray powder diffraction showed a pure orthorhombic phase, which was used for cell performance and other characterization. All heat treatments were carried out in air with a heating and cooling rate of 5°/min.

### Fabrication of the thin anode support and single cell^53^

Anode support (AS) materials, NiO (K ceracell, Korea) and BCZYZn05 powders (sintered @ 1400 °C), were mixed (65:35 wt%), ball-milled with 10 wt% corn starch (as a pore-former) in ethanol for 24 h, and then dried in an oven. These powders were pressed at 20 MPa for 1 min and then calcined at 900 °C for 2 h. For the anode functional layer (AFL) of the cells, NiO–BCZYZn05 powders were ball-milled with Solsperse (Lubrizol), polyvinylbutyral (PVB, Butvar, B-98), and di-n-butyl phthalate (DBP, Daejung Chemicals & Metals, 99%). The as-prepared AFL slurries were drop-coated onto the sintered anode pellet and then fired at 400 °C for 2 h. The BCZYZn05 powders were ball-milled with Solsperse, PVB, and DBP for 48 h. The resultant slurries were drop-coated onto the presintered anode substrate and then sintered at 1450 °C for 4 h. For the cathode materials, composite cathode inks were fabricated by mixing BSCF and BCZYZn05 in a weight ratio of 3:2 with terpineol using a mortar and pestle. The composite cathode ink was screen printed onto the composite electrolyte side (i.e., NiO-BCZYZn05 anode substrate|NiO-BCZYZn05 AFL|BCZYZn05 electrolyte|BSCF-BCZYZn05 composite cathode) and then fired at 1000 °C for 2 h. For the electrical measurements, platinum (Pt) paste (Heraeus, USA) was coated on both sides of the pellets and dried at 120 °C for 30 min in an oven. Au wires were used as the current collector and attached to the electrode using Pt paste.

### X-ray diffraction, neutron diffraction, SEM, EIS, TGA and nanoindentation

The phase purity of the material was investigated by X-ray diffraction analysis (XRD, D/MAX 2500, Rigaku, USA) using Cu-Kα_1_ (λ = 1.5406 Å) radiation in the 2θ range from 20° to 80°. Neutron diffraction data were collected using position-sensitive detectors in a neutron diffractometer at the Bangladesh Atomic Energy Commission. The neutron diffraction scan covered 2θ angles between 10° and 110°. The microstructure was measured by field emission scanning electron microscopy (FE-SEM, S-4700, Hitachi High tech, Japan). The anode and cathode sides of the single cell were fed H_2_ (3 vol% humidification) and dry air, respectively, at a flow rate of 200 sccm in the temperature range of 500–700 °C. Gas humidification was performed by bubbling air through a bottle with deionized water. Current–voltage–power (IVP) curves were measured using an SOFC test station (NARA Cell Tech Corp., Korea) using a potentiostat/galvanostat EIS instrument (SP-240, Biologic, Claix, France). The conductivity was determined by electrochemical impedance spectroscopy (EIS) using a potentiostat/galvanostat from 6 MHz to 0.1 Hz in the temperature range of 500–700 °C. An equivalent circuit model was fitted to the impedance spectra using EC-Lab software (provided from Biologic) to estimate the conductivity of the components. The weight change of the proton conductor was examined using the thermogravimetry (TG) technique by NETZSCH (STA 449F3, Germany). The sample was heated from room temperature to 900 °C at a heating rate of 10 °C min^−1^ under a N_2_ atmosphere.

A diamond Berkovich indenter tip was used to determine the hardness and elastic modulus of the anode-supported SOFC. It has a three-sided pyramidal shape. The Berkovich tip has a half angle of 65.27 degrees measured from the axis to one of the pyramid sides. Since it has a sharp and well-defined tip geometry, it is good for measuring the modulus and hardness value. However, the elastic–plastic transition may not be truly clear. The rate of force was fixed at 1 mN/s for both loading and unloading of the tip. Eight random locations were selected inside the center part for both samples, and eight random locations were selected in the outer part for both samples. The indentation points were perpendicular to the cell interface of the SOFC.

## Supplementary Information

Supplementary Information.
