# Supplementary material for: Improved mechanical strength, proton conductivity and power density in an ‘all-protonic’ ceramic fuel cell at intermediate temperature
Source: Sci Rep. 2021 Sep 29;11:19382. doi: 10.1038/s41598-021-98987-6 (PMC8481227; doi:10.1038/s41598-021-98987-6)
Supplement: Supplementary file 1 — Supplementary Information. [file 41598_2021_98987_MOESM1_ESM.docx]

Supplementary Materials for

**Improved mechanical strength, proton conductivity and power density in an ‘all-protonic’ ceramic fuel cell at intermediate temperature**

**Abul K. Azad^1,*^ Abdalla M. Abdalla^2^, Ahmed Afif ^1^, Atia Azad^3^, Shammya Afroze^1^, Azam Che Idris^1^, Jun-Young Park^4^, Mohammad Saqib^4^, Nikdalila Radenahmad^1^, Shahzad Hossain^5^, Iftakhar Bin Elius^5^,** **Md. Al-Mamun^5^, Juliana Zaini^1^, Amer Al-Hinai^6^, Md. Sumon Reza^1^, John T.S. Irvine^3^**

^1^Faculty of Integrated Technologies, Universiti Brunei Darussalam, JalanTungku Link, Gadong BE1410, Brunei Darussalam.

^2^Mechanical Engineering Department, Faculty of Engineering, Suez Canal University, Ismailia 41522, Egypt.

^3^School of Chemistry, University of St Andrews, Fife KY16 9ST, UK.

^4^HMC & Green Energy Research Institute, Department of Nanotechnology and Advanced Materials Engineering, Sejong University, Seoul 143-747, Republic of Korea.

^5^Institute of Nuclear Science and Technology, Bangladesh Atomic Energy Commission, Savar, Dhaka, Bangladesh.

^6^Sustainable Energy Research Center, Sultan Qaboos University, Muscat, Oman.

*Corresponding author: Email: [abul.azad@ubd.edu.bn](mailto:abul.azad@ubd.edu.bn) (A.K.A)

## Nano-indentation for Hardness test

Nano-indentation was performed using the following procedure:

- 1. Diamond Berkovich Indenter Tip was used for the test. It has a three-sided pyramidal shape. The Berkovich tip has a half abgle of 65.27 degrees measured from the axis to one of the pyramid sides. Since it has a sharp and well-defined tip geometry, it is good for measureming modulus and hardness value. However, elastic-plastic transition will not be truly clear.
  2. The rate of force was fixed at 1 mN/s for both loading and unloading of the tip.
  3. Eight random locations were selected inside the center part for both samples
  4. Eight random locations were selected inside the outer part for both samples
  5. The test can measure the Maximum depth, plastic depth, Hardness and Reduced Modulus (E_r_)
  6. The Young’s modulus for the sample (E_s_) can be calculated from the Reduced Modulus (E_r_) by using the relations below:
     -
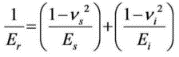

  7. For this diamond tip, the Young’s modulus for the tip (E_i_) and Poisson’s ratio for the tip (v_i_) are 1140 GPa and 0.07

Indentation results for the two samples are shown in Figs. S3-S6 and listed in Tables S2-S5.

**Electrochemical Impedance analysis**

The anode and cathode sides of the single cell were fed with H_2_ (3 vol% humidification) and dry air, respectively, at a flow rate of 200 sccm in the temperature range of 500 – 700℃. Gas humidification was performed by bubbling air through a bottle with de-ionized water. Current – voltage - power (IVP) curves were measured using an SOFC test station (NARA Cell Tech Corp., Korea) using a potentiostat/galvanostat EIS instrument (SP-240, Biologic, Claix, France).The conductivity was determined by electrochemical impedance spectroscopy (EIS) using a potentiostat/galvanostat from 6 MHz to 0.1 Hz in the temperature range of 500 – 700°C. An equivalent circuit model was fitted to the impedance spectra using the EC-Lab software (provided form Biologic) to estimate the conductivity of the components. The weight change of the proton conductor was examined using the thermogravimetry (TG) technique by NETZSCH (STA 449F3, Germany).The sample was heated from room temperature to 900°C at a heating rate of 10°C min^-1^ under N_2_ atmosphere.

To further illustrate the good performance of the single cell, electrochemical impedance spectra of the cell were obtained at various working temperatures. Typical Nyquist plots at different temperatures are shown in Figure S7. The high-frequency intercept on the real axis represents the ohmic resistance (R_ohmic)_ of the cell, which mainly originates from the resistance of the electrolyte, while the difference between the low-frequency and high-frequency intercepts with the real axis designates the interfacial polarization resistance (R_p_), which is mainly dominated by the electrode materials and microstructures^1^.

The area-specific resistance (ASR) of a single cell was calculated (fitted) from the impedance spectra by EC lab software with an equivalent circuit model, where R_1_ is R_ohmic_ and the sum of R_2_ and R_3_ is R_p_. Figure S7(b) presents the fitting profile of the obtained spectra with an equivalent circuit at 600°C. The obtained frequencies for the first semicircle and second semicircle were 2.3 kHz and 6.7 Hz, respectively. The conductivity values of the single cell were 2.3 × 10^-2^, 5.17 × 10^-2^, 0.13, 0.34 and 0.97 Scm^-1^ at 500, 550, 600, 650 and 700°C, respectively. Figure S7(c) exhibits the Arrhenius plot of the single cell. The bulk and total conductivities of the electrolyte material reached 9.23 × 10^-3^ and 7.61 × 10^-3^ underthe wet Ar condition, which were reported in a previous study^2^, where the activation energies were below 0.6 eV. Proton migration is the result of the incorporation of water into oxygen vacancies, generated by acceptor-doping the host ceramic material, which can be expressed in the Kröger–Vink notation using the equation (1).

$H_{2}O \left( g \right)+ v_{o}^{\cdot\cdot}+ O_{o}^{\times} = {2OH}_{o}^{\cdot}$ (1)

Protons are therefore introduced as point defects that become mobile as the temperature is increased, via the trans-port phenomenon known as the Grotthus mechanism^3^. This low activation energy makes fuel cells more proton conductive, affordable and practical.

The R_ohmic_ values were0.196, 0.238, 0.269, 0.266 and 0.306 Ωcm^2^ and the R_p_ values were0.07, 0.207, 0.543, 1.405 and 3.427 Ωcm^2^ at 700, 650, 600, 550 and 500°C, respectively. Figure S7 (d) shows that both R_ohmic_ and R_p_ increased remarkably with the decreasing operational temperature. Compared with previous results, both R_ohm_ and Rp values are lower than those of many BCZY-based cells^4^. The excellent performance of the present cell is due to the low cell resistances. The low R_ohmic_ values should be attributed to the highly dense and thin electrolyte film. Above 600°C, the R_p_ values were high due to the use of conventional BSCF. The cell performance can be improved more by lowering the R_p_ values using better cathode materials and reducing cell thickness. Moreover, the high performance combined with good conductivity demonstrates that the BaCe_0.7_Zr_0.1_Y_0.15_Zn_0.05_O_3-δ_ cell is a promising SOFC applicable at reduced temperatures.


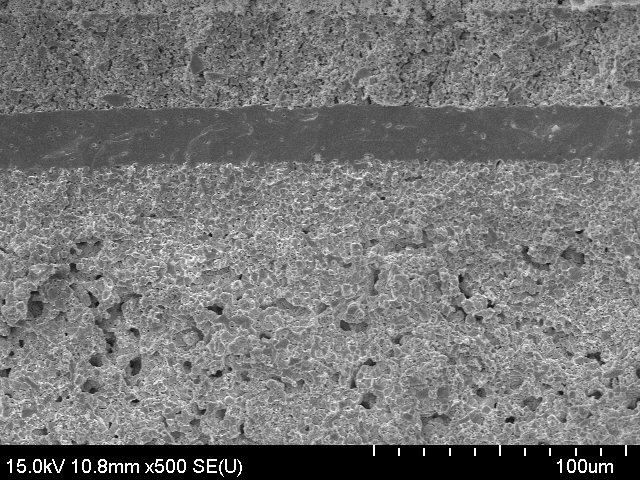

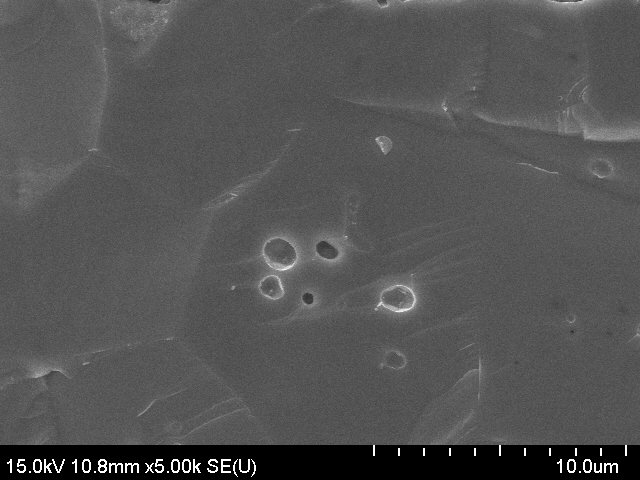

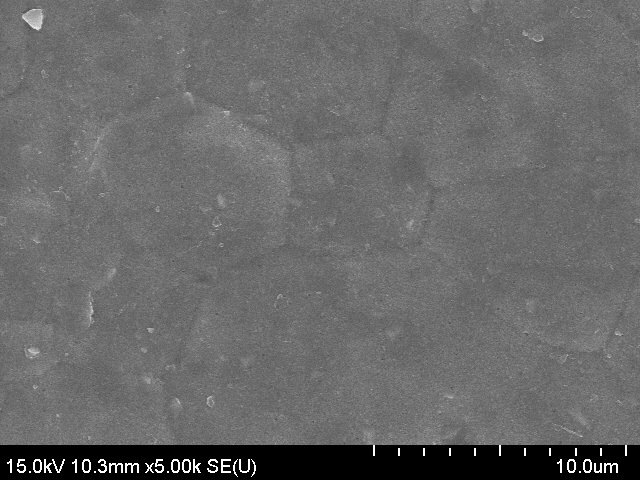


**(a)**

**(b)**

**Cathode (BSCF-BCZYZn05)**

**Electrolyte (BCZYZn05)**

**AFL (NiO-BCZYZn05)**

**Anode-support (NiO-BCZYZn05-Corn Starch)**


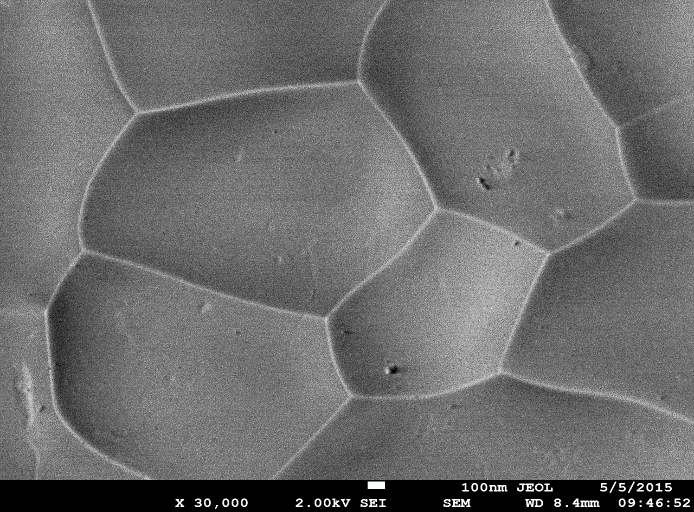


**(c)**

**(d)**

Figure S1.

The microstructure of the (a) cross-section of the single cell indicating porous anode and cathode, and dense electrolyte, (b) cross-section of the electrolyte (BCZYZn05), (c) surface of the electrolyte after fuel cell performance test and (d) surface of the as prepared BCZYZn05.


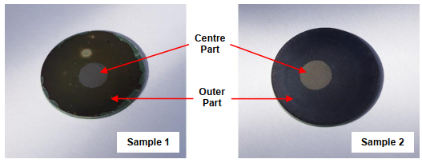


**Figure S2.**

**Multiple points selected inside centre part and outer part for both samples. Sample 1 (BSCF|BSCZYSm|Ni-BSCZYSm) and Sample 2 (BSCF-BCZYZn05 |BCZYZn05| Ni-BCZYZn)**


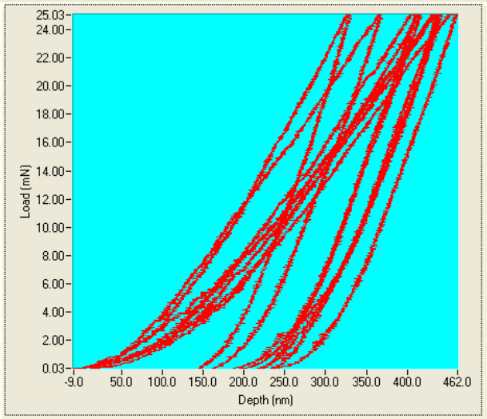


**Figure S3.**

**Depth vs Load for Sample 1 (Outer).**


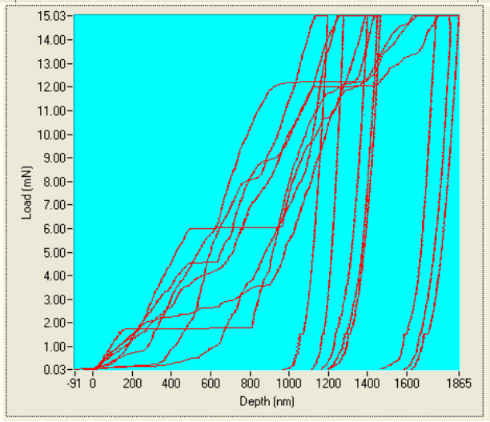


**Figure S4.**

**Depth vs Load for Sample 1 (Centre)**


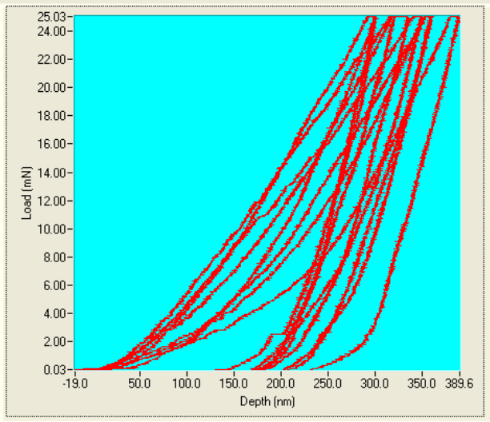


**Figure S5.**

**Depth vs Load for Sample 2 (Outer).**


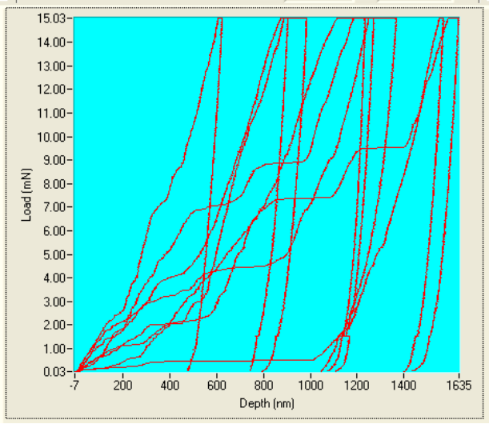


**Figure S6.**

**Depth vs Load for Sample 2 (Centre).**


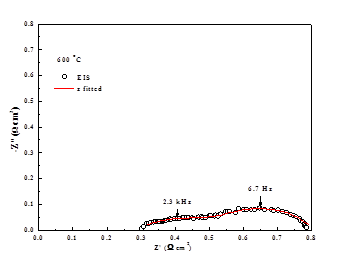

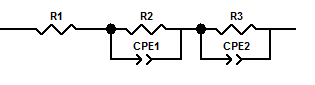


**(a)**

**(b)**

**(c)**

**(d)**

Figure S7.

(a) Electrochemical impedance spectra at 500 ~ 700°C, (b) Fitted Nyquist impedance plot at 600°C with equivalent circuit, (c) Arrhenius plot and (d) Ohmic resistance and polarization resistance of the single cell at different temperatures under open circuit conditions.

**A**


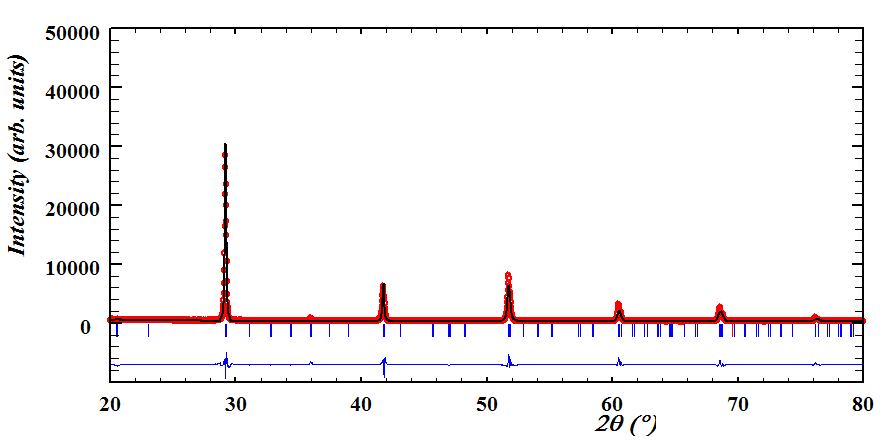


**b**


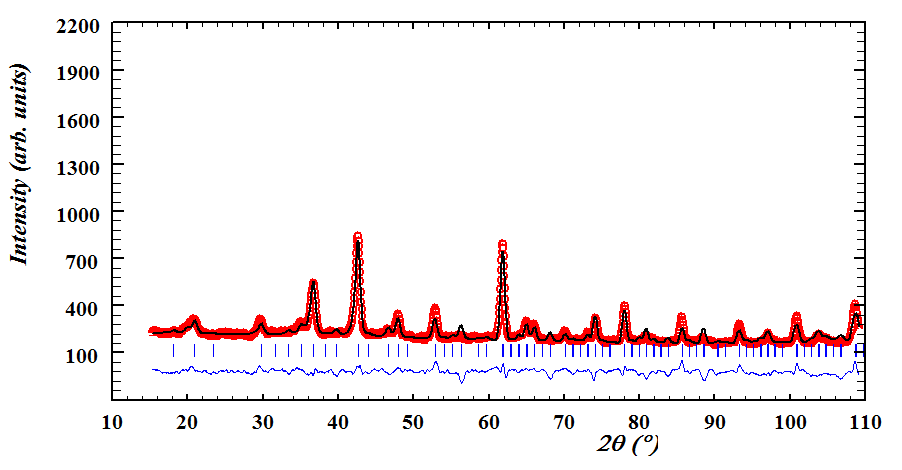


**Figure S8.**

**Rietveld refinement of the X-ray diffraction data (a) neutron diffraction data (b) of BCZYZn05 polycrystalline ceramic powder in orthorhombic symmetry in the Pbnm space group. The unit cell parameters are related to the ideal primitive cubic perovskite cell as a ≈ √2a_p_, b ≈ √2a_p_ and c ≈ 2a_p_ (a_p_ ≈ 3.96 Å is the unit cell parameter of ideal primitive perovskite).**

| **Sample Parameter** | | **XRD data of BCZYZn05** | **Neutron data of BCZYZn05** |
| --- | --- | --- | --- |
| *Space group* | | *Pbnm* | *Pbnm* |
| ***χ^2^*** | | 3.80 | 1.84 |
| *Cell parameter(Å)* | *a* | 6.228(2) | 6.162(1) |
|  | *b* | 6.256(2) | 6.149(1) |
|  | *c* | 8.782(3) | 8.714(2) |
| *Calculated density(g/cm^3^)* | | 6.675 | 6.653 |
| *Measured density(g/cm^3^)* | | 6.532 | 6.532 |
| *Relative density (phase 1) (%)* | | 97.86 | 98.18 |
| *Unit cell volume(Å^3^)* | | 342.014 | 330.201 |
| *No. of fitted parameters* | | 26 | 22 |
| *R_f_- factor (%)* | | 7.32 | 56.1 |
| *R_p_ (%)* | | 5.84 | 5.85 |
| *R_wp_ (%)* | | 7.54 | 7.44 |

**Table S1.**

**Rietveld refinement results for the XRD and neutron data for BCZYZn05**


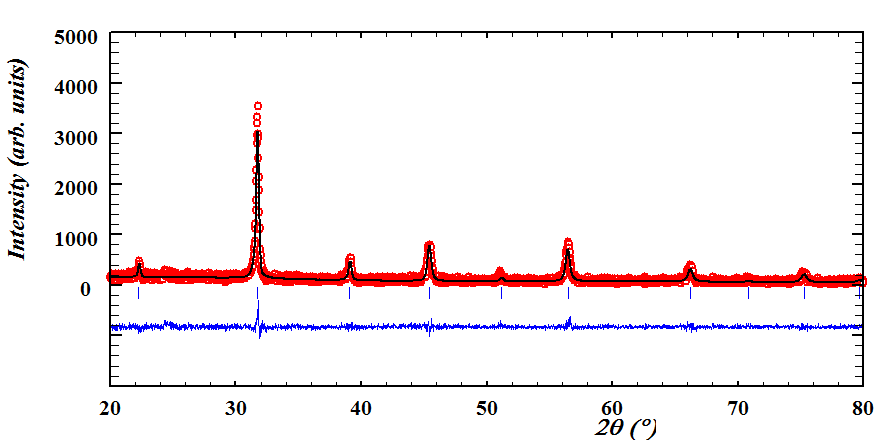


**Figure S9 Rietveld refinement of the X-ray diffraction data of BSCF.**

| **Sample 1 – Outer (on electrolyte)** | |  |  |  |  |
| --- | --- | --- | --- | --- | --- |
| **Indent** | **Max. Depth (nm)** | **Plastic Depth (nm)** | **Max. Load (mN)** | **Hardness (GPa)** | **Reduced Modulus (GPa)** |
| 1 | 331.6 | 236.3 | 25.03 | 13.38 | 127.7 |
| 2 | 462 | 352.9 | 25.03 | 6.731 | 79.072 |
| 3 | 417.8 | 300.4 | 25.03 | 8.902 | 84.513 |
| 4 | 439.6 | 340.8 | 25.03 | 7.154 | 90.001 |
| 5 | 443.3 | 326.5 | 25.03 | 7.708 | 79.034 |
| 6 | 369.7 | 260.5 | 25.03 | 11.356 | 102.641 |
| 7 | 439.1 | 330.8 | 25.03 | 7.534 | 84.252 |
| 8 | 412.7 | 303.5 | 25.03 | 8.745 | 90.092 |
| **Mean** | **414.5** | **306.5** | **25.03** | **8.939** | **92.163** |
| **Std. Dev** | **43.5** | **40.4** | **0** | **2.304** | **16.241** |

**Table S2.**

**Nano indentation measurement results for sample 1.**

| **Sample 1 – Centre (on cathode)** | |  |  |  |  |
| --- | --- | --- | --- | --- | --- |
| **Indent** | **Max. Depth (nm)** | **Plastic Depth (nm)** | **Max. Load (mN)** | **Hardness (GPa)** | **Reduced Modulus (GPa)** |
| 1 | 1453.4 | 1383.1 | 15.03 | 0.322 | 20.115 |
| 2 | 1466.9 | 1394.6 | 15.03 | 0.317 | 19.408 |
| 3 | 1279.3 | 1223.5 | 15.03 | 0.408 | 28.499 |
| 4 | 1749.2 | 1681.1 | 15.03 | 0.221 | 17.213 |
| 5 | 1865.4 | 1785.7 | 15.03 | 0.197 | 13.884 |
| 6 | 1197.6 | 1129.9 | 15.03 | 0.475 | 25.345 |
| 7 | 1823.3 | 1748.4 | 15.03 | 0.205 | 15.065 |
| 8 | 1400 | 1329.5 | 15.03 | 0.348 | 20.834 |
| **Mean** | **1529.4** | **1459.5** | **15.03** | **0.312** | **20.045** |
| **Std. Dev** | **252.6** | **247.9** | **0** | **0.1** | **4.948** |

**Table S3.**

**Nano indentation measurement results for sample 1.**

| **Sample 2 - Outer** | |  |  |  |  |
| --- | --- | --- | --- | --- | --- |
| **Indent** | **Max. Depth (nm)** | **Plastic Depth (nm)** | **Max. Load (mN)** | **Hardness (GPa)** | **Reduced Modulus (GPa)** |
| 1 | 361.6 | 286.1 | 25.03 | 9.679 | 132.495 |
| 2 | 350.4 | 283.4 | 25.03 | 9.837 | 150.702 |
| 3 | 389.6 | 320.9 | 25.03 | 7.941 | 132.07 |
| 4 | 321.1 | 249.4 | 25.03 | 12.226 | 156.685 |
| 5 | 335.8 | 268.8 | 25.03 | 10.766 | 157.441 |
| 6 | 302.7 | 249.3 | 25.03 | 12.229 | 210.706 |
| 7 | 361.1 | 299 | 25.03 | 8.977 | 155.092 |
| 8 | 300.4 | 243.2 | 25.03 | 12.752 | 200.751 |
| **Mean** | **340.3** | **275** | **25.03** | **10.551** | **161.993** |
| **Std. Dev** | **31.2** | **27.4** | **0** | **1.735** | **28.945** |

**Table S4.**

**Nano indentation measurement results for sample 2.**

| **Sample 2 – Centre** | |  |  |  |  |
| --- | --- | --- | --- | --- | --- |
| **Indent** | **Max. Depth (nm)** | **Plastic Depth (nm)** | **Max. Load (mN)** | **Hardness (GPa)** | **Reduced Modulus (GPa)** |
| 1 | 1570.6 | 1513.6 | 15.03 | 0.271 | 22.788 |
| 2 | 624.2 | 558.1 | 15.03 | 1.777 | 50.241 |
| 3 | 905 | 845.8 | 15.03 | 0.822 | 38.156 |
| 4 | 1635.2 | 1574.3 | 15.03 | 0.251 | 20.509 |
| 5 | 1368.9 | 1262.5 | 15.03 | 0.384 | 14.508 |
| 6 | 1235.7 | 1195.1 | 15.03 | 0.426 | 40.072 |
| 7 | 1272.1 | 1217 | 15.03 | 0.412 | 29.06 |
| 8 | 984.5 | 920.5 | 15.03 | 0.7 | 32.57 |
| **Mean** | **1199.5** | **1135.9** | **15.03** | **0.63** | **30.988** |
| **Std. Dev** | **343.7** | **343.4** | **0** | **0.104** | **11.707** |

**Table S5.**

**Nano indentation measurement results for sample 2.**

| **Geometry** | | | **Electrochemical properties** | | | | | **Reference** |
| --- | --- | --- | --- | --- | --- | --- | --- | --- |
| Cell component | Electrolyte integration method | Electrolyte thickness (μm) | OCV  (V) | R_ohm_ (Ω cm^2^) | R_p_ (Ω cm^2^) | Power Density  (mWcm^-2^) | Temperature  (^o^C) |  |
| NiO-BCZYZn05 \| BCZYZn05 \| BSCF-BCZYZn05 | drop coating | 26.6 | 1.04 | 0.196 | 0.07 | 872 | 700 | This work |
| NiO-BCZYZn05 \| BCZYZn05 \| BSCF-BCZYZn05 | drop coating | 26.6 | 1.027 | 0.238 | 0.207 | 670 | 650 | This work |
| NiO-BCZYZn05 \| BCZYZn05 \| BSCF-BCZYZn05 | drop coating | 26.6 | 1.037 | 0.269 | 0.543 | 445 | 600 | This work |
| NiO-BCZY3 \| BCZY3 \| BSCF | Screen printing | 5 | 1.06 | 0.09 |  | 1302 | 600 | ^5^ |
| NiO-BCZY712 \| BCZY712 \| SSC | tape casting | 17.6 | 1.04 |  |  | 750 | 600 | ^6^ |
| NiO-BCZY712 \| BCZY712 \| LSF-BCZY712 | co-pressing | 20 |  | 0.17  0.14 | 0.12  0.08 | 645  838 (micro-wave sintering) | 700 | ^7^ |
| NiO-BZCY352 \| BZCY352 \| SSC-SDC | co-pressing | 27 | 1.02 |  |  | 396 | 600 | ^4^ |
| NiO-BZCYZn04 \| BZCYZn04 \| SSC-BZCYZn04 | co-pressing | 20 | 1.05 | ~1.0 | ~0.95 | 246 | 600 | ^8^ |
| NiO-BZCYZn04 \| BZCYZn04 \| PBC-BZCYZn04 | co-pressing | 20 | 1.04 | ~0.75 | ~0.5 | 194 | 600 | ^9^ |
| NiO-BZCYZn04 \| BZCYZn04 \| LSCF-BZCYZn04 | tape casting | 25 | 1.02 | - | 1.12 | 175 | 600 | ^10^ |
| NiO-BZCYZn04 \| BZCYZn04 \| BSZF5528 | co-pressing | 30 | 1.06 |  |  | 122 | 600 | ^11^ |
| Ni-BZCYYb \| BZCYYb \| LSCF-BZCY | solution coating | 10 | > 1.0 |  |  | 1100 | 750 | ^12^ |
| Ni-BZCYYb \| BZCYYb \| LSCF-BZCYYb | particle suspension | 15 | 0.98 |  |  | 1020 | 700 | ^13^ |
| Ni-BZCYYb \| BZCYYb \| LSCF-BZCYYb | spin coating | 10 | 0.99 |  |  | 1000 | 750 | ^14^ |
| Ni-BZCYYb \| BZCYYb \| LSM-SDC | phase inversion | 12 | 0.93 |  |  | 890 | 700 | ^15^ |
| NiO-BZCYYb \| BZCYYb \| LSCF-BZCYYb | solution coating | 12 | 1.0 |  |  | 810 | 700 | ^16^ |
| NiO-BZCYYb4411\|BZCYYb4411\|PBSCF | Drop-casting, anode supported | 15 |  |  |  | 500 | 500 | ^17^ |
| NiO-BZY\|BZYNiO4\|PBCO | Co-pressing | 12 | 0.99 |  |  | 240 | 600 | ^18^ |
| *Full composition of the abbreviations* | BaCe_0.55_Zr_0.3_Y_0.15_O_3-δ_ (BCZY3), BaCe_0.7_Zr_0.1_Y_0.2_O_3-δ_ (BCZY712), Sm_0.5_Sr_0.5_CoO_3−δ_ (SSC), La_0.7_Sr_0.3_FeO_3−α_ (LSF), BaZr_0.3_Ce_0.5_Y_0.2_O_3−δ_ (BZCY352), BaZr_0.1_Ce_0.7_Y_0.1_Yb_0.1_O_3-δ_ (BZCYYb), BaCe_0.5_Zr_0.3_Y_0.16_Zn_0.04_O_3−δ_ (BZCYZn04), PrBaCo_2_O_5+δ_ (PBC), La_0.75_Sr_0.25_MnO_3−α_ (LSM), LaSr_3_Co_1.5_Fe_1.5_O_3−δ_ (LSCF), Ba_0.5_Sr_0.5_Zn_0.2_Fe_0.8_O_3−δ_ (BSZF), BaZr_0.4_Ce_0.4_Y_0.1_Yb_0.1_O_3_ (BZCYYb4411), PrBa_0.5_Sr_0.5_Co_1.5_Fe_0.5_O_5+d,_ BaZr_0.76_Y_0.2_Ni_0.04_O_3-δ_ (BZYNi04), PrBaCo_2_O_5+δ_ (PBCO) | | | | | | | |

**Table S6.**

**Comparison of electrochemical properties with those of PCFCs.**

# References

1. Sun, W., Liu, M. & Liu, W. Chemically stable yttrium and tin co-doped barium zirconate electrolyte for next generation high performance proton-conducting solid oxide fuel cells. *Adv. Energy Mater.* **3**, 1041–1050 (2013).

2. Afif, A. *et al.* Structural study and proton conductivity in BaCe0.7Zr0.25−xYxZn0.05O3 (x = 0.05, 0.1, 0.15, 0.2 &amp; 0.25). *Int. J. Hydrogen Energy* **41**, 11823–11831 (2016).

3. Kreuer, K. D., Dippel, T., Hainovsky, N. G. & Maier, J. Proton conductivity: compounds and their structural and chemical peculiarities. *Berichte der Bunsengesellschaft für Phys. Chemie* **96**, 1736–1742 (1992).

4. Shi, Z., Sun, W. & Liu, W. Synthesis and characterization of BaZr0.3Ce0.5Y 0.2-xYbxO3-δ proton conductor for solid oxide fuel cells. *J. Power Sources* **245**, 953–957 (2014).

5. An, H. *et al.* A 5 × 5 cm2 protonic ceramic fuel cell with a power density of 1.3 Wcm–2 at 600 °C. *Nat. Energy* **3**, 870–875 (2018).

6. Nien, S. H., Hsu, C. S., Chang, C. L. & Hwang, B. H. Preparation of BaZr_0.1_Ce_0.7_Y_0.2_O_3-δ_ based solid oxide fuel cells with anode functional layers by tape casting. *Fuel Cells* **11**, 178–183 (2011).

7. Xu, X., Bi, L. & Zhao, X. S. Highly-conductive proton-conducting electrolyte membranes with a low sintering temperature for solid oxide fuel cells. *J. Memb. Sci.* **558**, 17–25 (2018).

8. Ding, H., Xue, X., Liu, X. & Meng, G. High performance protonic ceramic membrane fuel cells (PCMFCs) with Sm0.5Sr0.5CoO3-δ perovskite cathode. *J. Alloys Compd.* **494**, 233–235 (2010).

9. Jin, M., Zhang, X., Qiu, Y. & Sheng, J. Layered PrBaCo 2O 5+δ perovskite as a cathode for proton-conducting solid oxide fuel cells. *J. Alloys Compd.* **494**, 359–361 (2010).

10. Zhang, S. *et al.* Stable BaCe0.5Zr0.3Y0.16Zn0.04O3-δ thin membrane prepared by in situ tape casting for proton-conducting solid oxide fuel cells. *J. Power Sources* **188**, 343–346 (2009).

11. Lin, B. *et al.* Stable, easily sintered BaCe0.5Zr0.3y 0.16Zn0.04O3-δ electrolyte-based protonic ceramic membrane fuel cells with Ba0.5Sr0.5Zn 0.2Fe0.8O3-δ perovskite cathode. *J. Power Sources* **183**, 479–484 (2008).

12. Yang, L. *et al.* Enhanced Sulfur and Coking Tolerance of a Mixed Ion Conductor for SOFCs: BaZr0.1Ce0.7Y0.2–xYbxO3–δ. *Science (80-. ).* **326**, 126–9 (2009).

13. Rainwater, B. H., Liu, M. & Liu, M. A more efficient anode microstructure for SOFCs based on proton conductors. *Int. J. Hydrogen Energy* **37**, 18342–18348 (2012).

14. Nguyen, N. T. Q. & Yoon, H. H. Preparation and evaluation of BaZr0.1Ce0.7Y 0.1Yb0.1O3-δ (BZCYYb) electrolyte and BZCYYb-based solid oxide fuel cells. *J. Power Sources* **231**, 213–218 (2013).

15. Chen, C. *et al.* High performance of anode supported BaZr0. 1Ce0. 7Y0. 1Yb0. 1O3-δ proton-conducting electrolyte micro-tubular cells with asymmetric structure for IT-SOFCs. *J. Electroanal. Chem.* **844**, 49–57 (2019).

16. Liu, M. *et al.* Anode-supported tubular SOFCs based on BaZr0.1Ce 0.7Y0.1Yb0.1O3 - δ electrolyte fabricated by dip coating. *Electrochem. commun.* **13**, 615–618 (2011).

17. Choi, S. *et al.* Exceptional power density and stability at intermediate temperatures in protonic ceramic fuel cells. *Nat. Energy* **3**, 202–210 (2018).

18. Shafi, S. P., Bi, L., Boulfrad, S. & Traversa, E. Y and Ni Co-Doped {BaZrO}3as a Proton-Conducting Solid Oxide Fuel Cell Electrolyte Exhibiting Superior Power Performance. *J. Electrochem. Soc.* **162**, F1498--F1503 (2015).
